# Prevalence of Pellagra

**Published:** 1914-07

**Authors:** 


Prevalence of Pellagra.—An interesting study was made of pellagra at the recent meeting of the American Medical Association. In Spartansburg county, South Carolina, according to reports, pellegra is most prevalent in the cotton mill villages. It attacks particularly the women and the children and the old people of both sexes. The disease appears to originate especially through proximity to, or association with, pre-existing cases of pellagra. It spreads most rapidly where insanitary methods of refuse disposal are in use. In other words, pellagra results oftenest from long hours of labor in poorly ventilated buildings, from a lack of proper nourishment, from uncleanliness, and from insanitary sewage disposal. It is therefore largely a disease that breeds best in poverty and in the conditions usually accompanying poverty.

